# Association between METS-IR and heart failure: a cross-sectional study

**DOI:** 10.3389/fendo.2024.1416462

**Published:** 2024-07-01

**Authors:** Xiaozhou Su, Chunli Zhao, Xianwei Zhang

**Affiliations:** Department of Cardiology, Minzu Affiliated Hospital of Guangxi Medical University, Nanning, Guangxi, China

**Keywords:** metabolic score for insulin resistance, heart failure, NHANES, nonlinear associations, cross-sectional study

## Abstract

**Background:**

Prior research has indicated the importance of insulin resistance in the development of heart failure (HF). The metabolic score for insulin resistance (METS-IR), a novel measure for assessing insulin resistance, has been found to be associated with cardiovascular disease (CVD). Nevertheless, the relationship between METS-IR and heart failure remains uncertain.

**Methods:**

This cross-sectional study collected data from the 2007–2018 National Health and Nutrition Examination Survey (NHANES). Multivariable logistic regression analysis and smoothing curve fitting were performed to explore the relationship between METS-IR and the risk of heart failure. Subgroup analysis and receiver operating characteristic (ROC) curve analysis were also conducted.

**Results:**

A total of 14772 patients were included, of whom 485 (3.28%) had heart failure. We observed a significant positive association between METS-IR and the risk of heart failure in a fully adjusted model (per 1-unit increment in METS-IR: OR: 2.44; 95% CI: 1.38, 4.32). Subgroup analysis and interaction tests revealed no significant influence on this relationship. A saturation effect and nonlinear relationship between METS-IR and heart failure risk were found using a smoothing curve fitting analysis. The relationship was represented by a J-shaped curve with an inflection point at 40.966.

**Conclusions:**

The results of our study indicated a J-shaped association between METS-IR and HF in adults in the United States. METS-IR may be a promising novel index for predicting the risk of heart failure. More longitudinal studies are needed to further verify causal relationships and validate the results in different classifications of heart failure populations.

## 1 Introduction

Heart failure affects approximately 65 million adults worldwide, and the incidence and prevalence are expected to continue increasing in the coming decades (1). Heart failure represents a significant global health problem, with high morbidity and mortality rates. It places a significant burden on healthcare systems and patients’ quality of life, leading to both health risks and economic pressure. Consequently, the timely detection and intervention of individuals at high risk of developing heart failure is of critical importance. The prognosis of heart failure is influenced by a number of factors, including age, gender, etiology, left ventricular ejection fraction, and comorbidities. Comorbidities have been demonstrated to have a significant impact on the development and progression of heart failure (2). Diabetes mellitus (DM) is a well-known risk factor that contributes to a poorer prognosis in heart failure and leads to increased hospitalizations and mortality rates (3). Type 2 diabetes mellitus (T2DM) and heart failure often co-occur, with approximately 15–25% of individuals with heart failure also having diabetes. In fact, the prevalence of heart failure in diabetics is four times higher than in the general population. According to reports, approximately 6% of individuals diagnosed with diabetes will develop heart failure at some point in their lives. The results of the Framingham Heart Study indicate that the occurrence of heart failure is two to five times more common in people with diabetes compared to healthy individuals, and that this is associated with poor outcomes (4).

Insulin resistance (IR) is a common feature observed in individuals with metabolic syndrome and T2DM. It is considered a key indicator of diabetes-related heart disease (DHD) (5), which encompasses coronary artery disease, autonomic heart disease, and diabetes cardiomyopathy (DCM) (6). DCM increases mortality in diabetes patients (7). Increasing evidence suggests that IR is the primary etiological factor in the development of nonischemic HF and post-ischemic HF (8–10). Several research studies have demonstrated that insulin resistance (IR) can affect blood circulation and the myocardium, myocardial fibrosis, cardiac hypertrophy, and ventricular remodeling. These effects contribute to the development of DHD and may ultimately lead to cardiac diastolic dysfunction and progression to HF (5).

Although the hyperinsulinemic-euglycemic clamp method (HEC) is considered to be the best for determining IR, it is not practical to use in clinical settings. Consequently, alternative non-insulin-based markers, including the homeostasis model assessment for insulin resistance (HOMA-IR), triglyceride-glucose (TyG) index, TyG-body mass index (TyG-BMI), and triglyceride to high-density lipoprotein cholesterol (TG/HDL-C) ratio, have been devised as replacements for evaluating IR (11, 12). Nevertheless, HOMA-IR also requires additional blood samples and analytical costs. Furthermore, there is conflicting evidence regarding the role of these indicators in the screening, diagnosis and prognosis of CHD, particularly in patients with coexisting various metabolic diseases.

The metabolic score for insulin resistance (METS-IR), which demonstrates a higher concordance with the HEC. A significant association between METS-IR and the development of T2DM (13), hypertension, and ischemic heart disease (14) in some studies. In comparison to other indicators, METS-IR is more effective for identifying individuals at high risk of CVD (15). However, previous studies only focused on the correlation between METS-IR and many CVDs, without examining its involvement in heart failure. In this study, we conducted a cross-sectional analysis using data from NHANES to examine the association between METS-IR and the risk of HF. In addition, we further explored the interactions and stratified confounders in the association between METS-IR and the risk of HF in different subgroups.

## 2 Materials and methods

### 2.1 Study population

The study utilized data from the NHANES, which is conducted by the National Center for Health Statistics (NCHS). NHANES is a comprehensive survey that aims to collect representative information on the health and nutrition of the non-institutionalized civilian population in the United States. To ensure a diverse sample, NHANES uses a stratified, multistage probability approach to select participants from across the country. The survey collects data through standardized in-home interviews, physical examinations, and laboratory tests carried out at mobile examination centers. All NHANES studies passed the National Center for Health Statistics (NCHS) Ethics Review Board, and written informed consent was obtained from all participants (https://www.cdc.gov/nchs/nhanes/irba98.htm).

**Figure 1:**
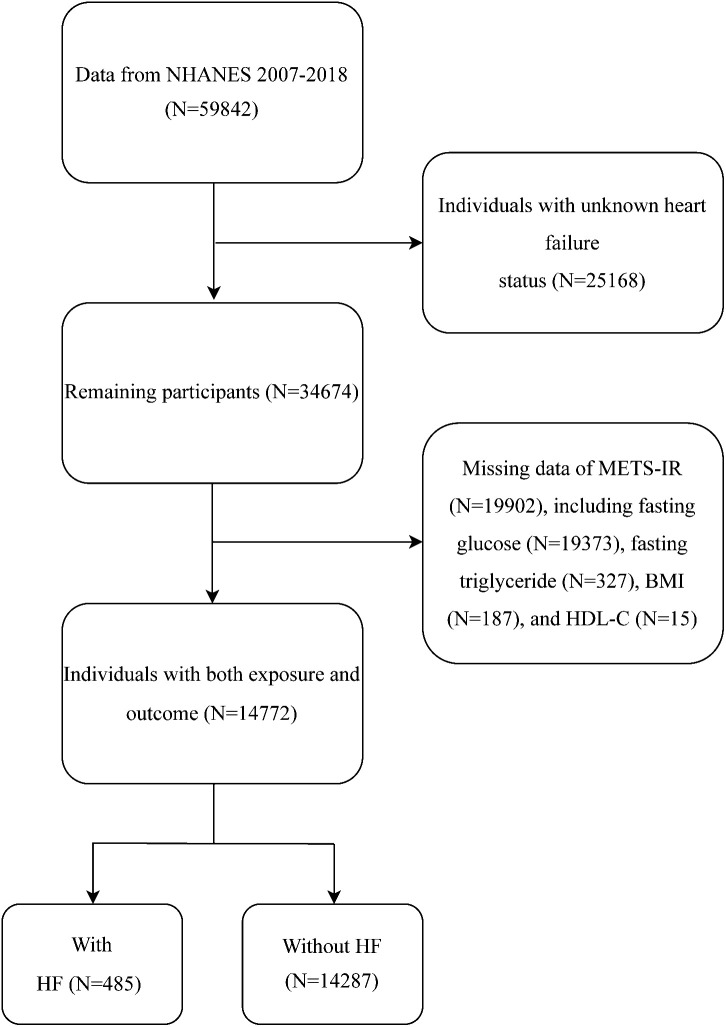
Flow chart of participant selection.

To explore the potential association between METS-IR and the risk of HF, our analysis was conducted using the 2007–2018 NHANES dataset. As shown in **Figure 1**, the initial sample consisted of 59842 participants. We then excluded participants who had no information on heart failure (n = 25168) and could not calculate METS-IR (n = 19902). A final total of 14772 participants were enrolled in this study.

### 2.2 Definitions of METS-IR and heart failure

METS-IR was calculated as ln [(2 × fasting plasma glucose (FPG) (mg/dL) + fasting triglycerides (TG) (mg/dL)] × body mass index (BMI) (kg/m^2^))/(ln[high-density lipoprotein cholesterol (HDL-c) (mg/dL]) (16). Subsequently, all participants were divided into four groups according to METS-IR quartiles: Group Q1 (<34.288), Group Q2 (34.288, 41.483), Group Q3 (41.483, 50.144), and Group Q4 (>50.144). Quality control of laboratory tests is available at https://wwwn.cdc.gov/nchs/nhanes/Default.aspx. Heart failure is defined in the study by asking participants a specific question regarding their medical history: “Have you ever been diagnosed with congestive heart failure by a doctor or other healthcare professional?” If participants respond affirmatively, their response is recorded as indicating the presence of congestive heart failure.

### 2.3 Covariates

Our study also selected possible factors that may affect the association between clinical relevance based METS-IR and heart failure, including age (year), gender (male/female), race (Mexican American/Other Hispanic/Non-Hispanic White/Non-Hispanic Black/Other Race), education level (below high school/high school or equivalent/college or above), marital status (married/unmarried), smoking status, drinking status, physical activity (PA), family poverty income ratio (PIR), waist circumference, body mass index (BMI, kg/m^2^), systolic blood pressure (SBP), diastolic blood pressure (DBP), fasting glucose, LDL-c (low-density lipoprotein cholesterol), total cholesterol (TC), hemoglobin (Hb), hemoglobin A1c (HbA1c), blood urea nitrogen (BUN), serum creatinine, eGFR (estimated glomerular filtration rate), serum uric acid, hypertension, high cholesterol, diabetes, coronary heart disease (CHD), angina pectoris, heart attack, stroke. Smoking status was grouped into “never” (never smoked or less than a hundred cigarettes in life), “current” (more than a hundred cigarettes in life and is also ongoing currently), or “former” (more than a hundred cigarettes in life but currently not smoking). Drinking status was grouped into “never” (never consumed alcohol more than 12 times in a year), “current” (more than 12 times in a year and is also ongoing currently), or “former” (consumed alcohol more than 12 times in a year but currently not drinking). Physical activity (PA) was determined according to answers to a survey about whether a subject was vigorously or moderately active in recreational activities. Hypertension was defined as a systolic blood pressure ≥ 140 mmHg or diastolic blood pressure ≥ 90 mmHg, or a self-reported history of hypertension or oral antihypertensive medications. High cholesterol was defined as a fasting TC ≥ 240 mg/dL or taking lipid-lowering agents. For diabetes, we adopted a comprehensive definition encompassing a fasting blood glucose level ≥126 mg/dL, a HbA1c ≥ 6.5%, use of oral hypoglycemic agents, insulin use, or self-reported history of diabetes. The formula used to eGFR was the Chronic Kidney Disease Epidemiology Collaboration (CKD-EPI) equation (17). All detailed measurement processes of the study variables are publicly available at www.cdc.gov/nchs/nhanes/.

### 2.4 Statistical analysis

Statistical analysis of the current study was rigorously conducted according to the design recommended by NHANES, taking into account sample weights, clustering, and stratified analysis. We used the weights of the fasting subsample and adjusted the weights to account for multiple cycles. Since we merged six cycles of NHANES datasets for data from 2007 to 2018, the SAF sample weights (WTSAF2YR/6) were applied for weighted analyses (https://wwwn.cdc.gov/nchs/nhanes/tutorials/default.aspx). Continuous variables were expressed as means ± standard deviations, and categorical variables were expressed as percentages. Based on their METS-IR levels, the study participants were divided into four groups or quartiles. The Chi-squared test or Kruskal-Wallis H test was employed to compare differences in population characteristics by METS-IR quartiles. In order to investigate the relationship between METS-IR and HF, a multivariate logistic regression analysis was used in the analysis to explore the relationship between METS-IR and HF. Depending on the adjustment for covariates, four models were generated. Model 1: no adjustment for covariates; Model 2: age, gender, and race were adjusted; Model 3: adjusted for covariates in Model 2 plus education level, marital status, smoking status, drinking status, hypertension, high cholesterol, CHD, angina, heart attack, stroke, and diabetes status; Model 4: adjusted for covariates in Model 3 plus PIR, PA, SBP, DBP, BUN, serum creatinine, eGFR, serum uric acid, HbA1c, Hb, TC, LDL-C, waist circumference. Subgroup analyses were carried out to examine the potential impacts of gender, age, BMI, eGFR, education level, smoking status, drinking status, CHD, hypertension, and diabetes status on the relationship between METS-IR and HF. In addition, smoothed curve fitting and threshold effect models in this study were used to verify the existence of non-linear relationships between the independent and response variables. A non-linear effects model was used when the likelihood ratio (LLR) was <0.05. The statistical analyzes of this study were performed via R, version 4.2.0 (R Foundation) and EmpowerStats (http://www.empowerstats.com, X&Y Solutions, Inc., Boston, MA). Statistical significance was determined by two-sided p values less than 0.05.

## 3 Results

### 3.1 Baseline characteristics

A total of 14772 participants were included in this study (**Figure 1**). Among them, 485 (3.28%) had heart failure. The mean age (95% CI) was 47.65 (47.18, 48.11) years, and the mean METS-IR was 43.11 (42.70, 43.52). As shown in **Table 1**, compared to non-HF individuals, the included HF population was more likely to be older, smokers, have hypertension, high cholesterol, diabetes, CHD, angina, heart attack, and stroke, individuals with lower education levels, income levels, and eGFR, and higher waist circumference, body mass index, SBP, fasting glucose, and HbA1c (p < 0.001). Notably, the HF group exhibited a significantly higher METS-IR than the non-HF group (48.58 vs. 42.92, p < 0.001).

**Table 1:** Weighted baseline characteristics of the HF and non-HF groups.

Variable^a^	Overall	Heart failure	Non-heart failure	P-value
Participants	14772	485	14287	
Age (year)	47.65 (47.18,48.11)	65.83 (64.18,67.48)	47.15 (46.69,47.62)	<0.001
Gender (%)				0.381
Male	48.52 (47.58,49.46)	51.81 (44.39,59.15)	48.43 (47.45,49.42)	
Female	51.48 (50.54,52.42)	48.19 (40.85,55.61)	51.57 (50.58,52.55)	
Race (%)				0.031
Mexican American	9.63 (8.35,11.08)	6.13 (3.72,9.91)	9.72 (8.43,11.19)	
Other hispanic	6.65 (5.73,7.70)	5.37 (3.08,9.19)	6.68 (5.76,7.74)	
Non-Hispanic White	62.75 (60.09,65.34)	65.45 (59.42,71.02)	62.68 (60.03,65.26)	
Non-Hispanic Black	12.24 (10.97,13.64)	16.75 (12.59,21.95)	12.12 (10.86,13.51)	
Other Race	8.73 (7.76,9.80)	6.31 (3.64,10.71)	8.79 (7.82,9.87)	
Education level (%)				<0.001
Less than high school	5.93 (5.34,6.58)	13.61 (10.07,18.16)	5.72 (5.11,6.39)	
High school or equivalent	11.17 (10.21,12.21)	18.21 (13.30,24.42)	10.98 (10.03,12.01)	
College or above	82.90 (81.64,84.10)	68.18 (61.31,74.33)	83.30 (82.04,84.49)	
Marital status (%)				0.017
Married	63.15 (61.70,64.58)	55.64 (49.21,61.89)	63.35 (61.87,64.81)	
Unmarried	36.85 (35.42,38.30)	44.36 (38.11,50.79)	36.65 (35.19,38.13)	
Smoker (%)				<0.001
Current	18.76 (17.61,19.96)	22.05 (16.53,28.78)	18.67 (17.51,19.88)	
Former	34.40 (32.87,35.98)	46.43 (40.47,52.49)	34.08 (32.51,35.69)	
Never	46.84 (45.02,48.66)	31.52 (26.10,37.50)	47.25 (45.43,49.08)	
Drinking status (%)				<0.001
Current	70.97 (69.42,72.48)	46.93 (41.03,52.92)	71.63 (70.10,73.11)	
Former	10.24 (9.44,11.09)	27.11 (21.86,33.10)	9.78 (9.01,10.60)	
Never	18.79 (17.57,20.07)	25.96 (21.09,31.49)	18.59 (17.36,19.89)	
PA (moderate, vigorous)				<0.001
Yes	72.51 (71.33,73.65)	50.65 (44.25,57.04)	73.10 (71.95,74.22)	
No	27.49 (26.35,28.67)	49.35 (42.96,55.75)	26.90 (25.78,28.05)	
PIR	2.83 (2.76,2.91)	2.13 (1.92,2.33)	2.85 (2.78,2.93)	<0.001
Waist circumference (cm)	99.28 (98.75,99.81)	106.08 (103.62,108.53)	99.10 (98.57,99.62)	<0.001
Weight (kg)	82.91 (82.32,83.50)	87.99 (84.69,91.30)	82.77 (82.17,83.37)	0.003
Height (cm)	168.70 (168.44,168.95)	167.38 (166.00,168.76)	168.73 (168.48,168.99)	0.061
BMI, kg/m^2^	29.05 (28.84,29.26)	31.25 (30.19,32.31)	28.99 (28.79,29.20)	<0.001
SBP (mmHg)	121.32 (120.88,121.76)	127.47 (125.32,129.62)	121.15 (120.72,121.59)	<0.001
DBP (mmHg)	69.80 (69.36,70.23)	66.66 (65.08,68.23)	69.88 (69.45,70.31)	<0.001
Fasting glucose (mg/dL)	106.59 (105.87,107.31)	120.16 (115.20,125.12)	106.22 (105.52,106.93)	<0.001
HDL-c (mg/dL)	54.22 (53.72,54.72)	49.40 (47.49,51.32)	54.35 (53.85,54.85)	<0.001
LDL-c (mg/dL)	113.62 (112.74,114.50)	103.31 (97.86,108.75)	113.90 (113.00,114.80)	<0.001
TG (mg/dL)	122.29 (119.75,124.84)	146.60 (130.56,162.65)	121.63 (119.11,124.15)	0.003
TC (mg/dL)	191.89 (190.73,193.06)	175.75 (169.78,181.72)	192.33 (191.12,193.54)	<0.001
METS-IR	43.11 (42.70,43.52)	48.58 (46.42,50.74)	42.96 (42.55,43.36)	<0.001
Hb (g/dL)	14.29 (14.23,14.34)	13.77 (13.57,13.98)	14.30 (14.25,14.35)	<0.001
HbA1c (%)	5.63 (5.61,5.66)	6.23 (6.07,6.39)	5.62 (5.60,5.64)	<0.001
BUN (mg/dL)	13.61 (13.47,13.75)	19.38 (18.20,20.56)	13.46 (13.32,13.60)	<0.001
Serum creatinine (mg/dL)	0.88 (0.87,0.89)	1.17 (1.10,1.24)	0.87 (0.86,0.88)	<0.001
eGFR (mL/min/1.73 m^2^)	90.95 (90.35,91.55)	73.34 (68.67,78.01)	91.43 (90.82,92.04)	<0.001
Serum uric acid (mg/dL)	5.48 (5.44,5.52)	6.42 (6.20,6.65)	5.45 (5.42,5.49)	<0.001
Hypertension (%)				<0.001
Yes	38.41 (37.10,39.74)	84.13 (78.99,88.20)	37.17 (35.88,38.48)	
No	61.59 (60.26,62.90)	15.87 (11.80,21.01)	62.83 (61.52,64.12)	
High cholesterol (%)				<0.001
Yes	43.54 (42.40,44.68)	71.48 (65.29,76.95)	42.78 (41.63,43.94)	
No	56.46 (55.32,57.60)	28.52 (23.05,34.71)	57.22 (56.06,58.37)	
Diabetes (%)				<0.001
Yes	14.55 (13.71,15.42)	43.98 (38.50,49.62)	13.75 (12.93,14.60)	
No	85.45 (84.58,86.29)	56.02 (50.38,61.50)	86.25 (85.40,87.07)	
CHD (%)				<0.001
Yes	4.15 (3.68,4.68)	40.09 (33.64,46.91)	3.18 (2.80,3.61)	
No	95.85 (95.32,96.32)	59.91 (53.09,66.36)	96.82 (96.39,97.20)	
Angina (%)				<0.001
Yes	2.25 (1.95,2.59)	23.49 (18.58,29.23)	1.67 (1.40,2.00)	
No	97.75 (97.41,98.05)	76.51 (70.77,81.42)	98.33 (98.00,98.60)	
Heart attack (%)				<0.001
Yes	3.69 (3.26,4.18)	43.52 (37.44,49.81)	2.61 (2.25,3.03)	
No	96.31 (95.82,96.74)	56.48 (50.19,62.56)	97.39 (96.97,97.75)	
Stroke (%)				<0.001
Yes	3.04 (2.69,3.44)	22.81 (18.35,27.99)	2.51 (2.18,2.88)	
No	96.96 (96.56,97.31)	77.19 (72.01,81.65)	97.49 (97.12,97.82)	

a Data were summarized as mean (95% confidence intervals) or percentage (95% confidence intervals) according to their data type.PA, physical activity; PIR, family poverty income ratio; BMI, body mass index; SBP, systolic blood pressure; DBP, diastolic blood pressure; HDL-c, high-density lipoprotein cholesterol; LDL-c, low-density lipoprotein cholesterol; TG, triglycerides; TC, total cholesterol; METS-IR, metabolic score for insulin resistance; Hb, hemoglobin; HbA1c, hemoglobin A1c; BUN, blood urea nitrogen; eGFR, estimated glomerular filtration rate; CHD, coronary heart disease.

**Table 2** displays the participants’ clinical characteristics according to METS-IR quartiles. The values for the different quartiles were as follows: quartile 1 (<34.288), quartile 2 (34.288, 41.483), quartile 3 (41.483, 50.144), and quartile 4 (>50.144). Compared to participants in the lower METS-IR group, participants in the METS-IR Q4 group were more likely to be Mexican Americans, smokers, hypertension, high cholesterol, diabetes, CHD, angina, heart attack, individuals with lower education levels, less physical activity, higher levels of waist circumference, weight, BMI, blood pressure, fasting glucose, TG, HbA1c, serum uric acid, and lower HDL-c (all P < 0.01). Importantly, participants with high levels of METS-IR had a higher prevalence of HF (P < 0.01).

**Table 2:** Weighted baseline characteristics of participants by quartiles of baseline METS-IR.

Variable^a^	Overall	METS-IR Quartiles				P-value
		Q1 (<34.288)	Q2 (34.288–41.483)	Q3 (41.483–50.144)	Q4 (>50.144)	
Age (year)	47.65 (47.18,48.11)	44.81 (43.84,45.77)	48.51 (47.73,49.29)	49.57 (48.79,50.35)	47.93 (47.18,48.67)	<0.001
Gender (%)						<0.001
Male	48.52 (47.58,49.46)	38.48 (36.37,40.64)	51.17 (49.01,53.34)	54.84 (52.79,56.87)	50.42 (48.37,52.47)	
Female	51.48 (50.54,52.42)	61.52 (59.36,63.63)	48.83 (46.66,50.99)	45.16 (43.13,47.21)	49.58 (47.53,51.63)	
Race (%)						<0.001
Mexican American	9.63 (8.35,11.08)	6.97 (5.97,8.13)	8.64 (7.22,10.31)	10.99 (9.30,12.93)	12.15 (10.24,14.37)	
Other hispanic	6.65 (5.73,7.70)	6.21 (5.05,7.61)	6.63 (5.50,7.97)	7.38 (6.08,8.93)	6.41 (5.40,7.60)	
Non-Hispanic White	62.75 (60.09,65.34)	63.78 (60.82,66.65)	62.73 (59.40,65.93)	61.60 (58.43,64.67)	62.82 (59.36,66.14)	
Non-Hispanic Black	12.24 (10.97,13.64)	11.02 (9.66,12.54)	12.65 (10.99,14.51)	12.20 (10.63,13.97)	13.20 (11.38,15.26)	
Other Race	8.73 (7.76,9.80)	12.02 (10.49,13.75)	9.36 (7.81,11.18)	7.84 (6.49,9.44)	5.42 (4.51,6.50)	
Education level (%)						<0.001
Less than high school	5.93 (5.34,6.58)	3.78 (3.11,4.58)	6.14 (5.29,7.12)	7.12 (6.11,8.30)	6.84 (5.88,7.94)	
High school or equivalent	11.17 (10.21,12.21)	10.39 (8.94,12.05)	10.19 (8.86,11.70)	11.09 (9.70,12.66)	13.06 (11.66,14.61)	
College or above	82.90 (81.64,84.10)	85.83 (83.87,87.59)	83.66 (81.85,85.33)	81.78 (79.86,83.56)	80.10 (78.43,81.67)	
Marital status (%)						0.001
Married	63.15 (61.70,64.58)	59.89 (57.21,62.50)	62.84 (60.38,65.23)	66.50 (64.16,68.77)	63.67 (61.27,66.01)	
Unmarried	36.85 (35.42,38.30)	40.11 (37.50,42.79)	37.16 (34.77,39.62)	33.50 (31.23,35.84)	36.33 (33.99,38.73)	
Smoker (%)						<0.001
Current	18.76 (17.61,19.96)	19.72 (17.60,22.02)	18.99 (17.04,21.10)	17.54 (15.97,19.24)	18.69 (17.02,20.49)	
Former	34.40 (32.87,35.98)	30.76 (28.18,33.47)	33.19 (30.57,35.91)	37.61 (35.49,39.78)	36.39 (33.84,39.02)	
Never	46.84 (45.02,48.66)	49.52 (46.74,52.31)	47.82 (45.04,50.62)	44.85 (42.53,47.19)	44.92 (42.34,47.52)	
Drinking status (%)						<0.001
Current	70.97 (69.42,72.48)	75.27 (73.05,77.36)	73.19 (70.78,75.48)	69.37 (66.92,71.72)	65.69 (63.20,68.10)	
Former	10.24 (9.44,11.09)	7.15 (5.95,8.57)	9.26 (8.18,10.47)	11.60 (10.17,13.20)	13.20 (11.62,14.97)	
Never	18.79 (17.57,20.07)	17.58 (15.97,19.31)	17.55 (15.69,19.57)	19.03 (17.36,20.81)	21.11 (19.09,23.28)	
PA (moderate, vigorous)						<0.001
Yes	72.51 (71.33,73.65)	76.96 (74.88,78.91)	74.45 (72.51,76.29)	71.18 (69.20,73.08)	67.07 (64.84,69.22)	
No	27.49 (26.35,28.67)	23.04 (21.09,25.12)	25.55 (23.71,27.49)	28.82 (26.92,30.80)	32.93 (30.78,35.16)	
PIR	2.83 (2.76,2.91)	2.94 (2.83,3.04)	2.95 (2.85,3.05)	2.82 (2.72,2.92)	2.62 (2.52,2.71)	<0.001
Waist circumference (cm)	99.28 (98.75,99.81)	82.31 (81.92,82.70)	94.35 (93.94,94.75)	103.25 (102.88,103.62)	118.60 (117.91,119.30)	<0.001
Weight (kg)	82.91 (82.32,83.50)	62.31 (61.91,62.72)	75.93 (75.45,76.41)	86.63 (86.11,87.14)	108.44 (107.53,109.35)	<0.001
Height (cm)	168.70 (168.44,168.95)	167.56 (167.12,168.00)	168.98 (168.51,169.46)	169.20 (168.74,169.65)	169.14 (168.66,169.63)	<0.001
BMI, kg/m^2^	29.05 (28.84,29.26)	22.13 (22.03,22.23)	26.53 (26.42,26.63)	30.22 (30.10,30.34)	37.90 (37.59,38.20)	<0.001
SBP (mmHg)	121.32 (120.88,121.76)	116.73 (115.98,117.47)	120.68 (119.95,121.41)	122.83 (122.19,123.47)	125.42 (124.75,126.08)	<0.001
DBP (mmHg)	69.80 (69.36,70.23)	67.34 (66.76,67.92)	68.93 (68.39,69.47)	70.81 (70.20,71.41)	72.32 (71.73,72.91)	<0.001
Fasting glucose (mg/dL)	106.59 (105.87,107.31)	95.84 (95.29,96.39)	101.80 (101.11,102.50)	108.24 (107.07,109.41)	121.37 (119.48,123.26)	<0.001
HDL-c (mg/dL)	54.22 (53.72,54.72)	67.68 (66.73,68.62)	56.08 (55.36,56.80)	48.81 (48.37,49.25)	43.20 (42.73,43.67)	<0.001
LDL-c (mg/dL)	113.62 (112.74,114.50)	105.59 (104.15,107.03)	117.39 (115.76,119.01)	118.63 (117.08,120.18)	113.52 (112.01,115.03)	<0.001
TG (mg/dL)	122.29 (119.75,124.84)	76.55 (74.58,78.52)	104.48 (101.93,107.03)	132.67 (129.01,136.33)	179.24 (172.04,186.44)	<0.001
TC (mg/dL)	191.89 (190.73,193.06)	188.55 (186.76,190.34)	194.46 (192.48,196.44)	194.18 (192.24,196.12)	190.65 (188.59,192.71)	<0.001
METS-IR	43.11 (42.70,43.52)	29.56 (29.43,29.69)	37.85 (37.76,37.95)	45.49 (45.38,45.59)	60.64 (60.12,61.16)	<0.001
Hb (g/dL)	14.29 (14.23,14.34)	14.03 (13.95,14.10)	14.32 (14.23,14.40)	14.44 (14.37,14.51)	14.38 (14.30,14.46)	<0.001
HbA1c (%)	5.63 (5.61,5.66)	5.34 (5.32,5.37)	5.49 (5.46,5.51)	5.67 (5.64,5.71)	6.06 (6.00,6.12)	<0.001
BUN (mg/dL)	13.61 (13.47,13.75)	12.96 (12.77,13.15)	13.62 (13.38,13.87)	14.00 (13.70,14.30)	13.92 (13.63,14.21)	<0.001
Serum creatinine (mg/dL)	0.88 (0.87,0.89)	0.84 (0.83,0.85)	0.89 (0.87,0.90)	0.89 (0.88,0.91)	0.89 (0.87,0.91)	<0.001
eGFR (mL/min/1.73 m^2^)	90.95 (90.35,91.55)	93.05 (91.92,94.18)	89.93 (89.00,90.86)	90.09 (89.07,91.12)	90.58 (89.62,91.54)	<0.001
Serum uric acid (mg/dL)	5.48 (5.44,5.52)	4.85 (4.79,4.91)	5.34 (5.28,5.39)	5.74 (5.68,5.81)	6.04 (5.98,6.10)	<0.001
Hypertension (%)						<0.001
Yes	38.41 (37.10,39.74)	23.63 (21.52,25.88)	35.34 (33.22,37.52)	41.99 (39.77,44.26)	53.88 (51.43,56.31)	
No	61.59 (60.26,62.90)	76.37 (74.12,78.48)	64.66 (62.48,66.78)	58.01 (55.74,60.23)	46.12 (43.69,48.57)	
High cholesterol (%)						<0.001
Yes	43.54 (42.40,44.68)	33.72 (31.53,35.99)	45.45 (43.09,47.84)	46.72 (44.69,48.75)	49.04 (46.84,51.24)	
No	56.46 (55.32,57.60)	66.28 (64.01,68.47)	54.55 (52.16,56.91)	53.28 (51.25,55.31)	50.96 (48.76,53.16)	
Diabetes (%)						<0.001
Yes	14.55 (13.71,15.42)	4.49 (3.70,5.45)	9.47 (8.20,10.92)	15.93 (14.53,17.44)	29.12 (27.28,31.03)	
No	85.45 (84.58,86.29)	95.51 (94.55,96.30)	90.53 (89.08,91.80)	84.07 (82.56,85.47)	70.88 (68.97,72.72)	
CHD (%)						0.001
Yes	4.15 (3.68,4.68)	2.94 (2.25,3.85)	3.71 (2.94,4.68)	4.69 (3.91,5.61)	5.37 (4.36,6.61)	
No	95.85 (95.32,96.32)	97.06 (96.15,97.75)	96.29 (95.32,97.06)	95.31 (94.39,96.09)	94.63 (93.39,95.64)	
Angina (%)						<0.001
Yes	2.25 (1.95,2.59)	1.47 (1.00,2.15)	1.67 (1.27,2.20)	2.50 (1.97,3.18)	3.42 (2.66,4.40)	
No	97.75 (97.41,98.05)	98.53 (97.85,99.00)	98.33 (97.80,98.73)	97.50 (96.82,98.03)	96.58 (95.60,97.34)	
Heart attack (%)						<0.001
Yes	3.69 (3.26,4.18)	2.63 (1.94,3.54)	3.48 (2.77,4.38)	3.86 (3.26,4.57)	4.89 (4.07,5.86)	
No	96.31 (95.82,96.74)	97.37 (96.46,98.06)	96.52 (95.62,97.23)	96.14 (95.43,96.74)	95.11 (94.14,95.93)	
Stroke (%)						0.019
Yes	3.04 (2.69,3.44)	2.61 (1.89,3.60)	3.03 (2.39,3.84)	2.54 (2.03,3.18)	4.03 (3.32,4.88)	
No	96.96 (96.56,97.31)	97.39 (96.40,98.11)	96.97 (96.16,97.61)	97.46 (96.82,97.97)	95.97 (95.12,96.68)	
Heart failure (%)						<0.001
Yes	2.64 (2.30,3.02)	1.81 (1.28,2.54)	1.90 (1.44,2.50)	2.43 (1.89,3.12)	4.48 (3.70,5.43)	
No	97.36 (96.98,97.70)	98.19 (97.46,98.72)	98.10 (97.50,98.56)	97.57 (96.88,98.11)	95.52 (94.57,96.30)	

a Data were summarized as mean (95% confidence intervals) or percentage (95% confidence intervals) according to their data type.PA, physical activity; PIR, family poverty income ratio; BMI, body mass index; SBP, systolic blood pressure; DBP, diastolic blood pressure; HDL-C, high-density lipoprotein cholesterol; LDL-C, low-density lipoprotein cholesterol; TG, triglycerides; TC, total cholesterol; METS-IR, metabolic score for insulin resistance; HbA1c, hemoglobin A1c; BUN, blood urea nitrogen; eGFR, estimated glomerular filtration rate; CHD, coronary heart disease.

### 3.2 Association between METS-IR and HF

The continuous variables analysis showed a positive association. For each unit increase (1%) in METS-IR, the risk of heart failure increased by 3% (OR = 1.03, 95% CI: 1.02–1.04) in the unadjusted model and in the fully adjusted model by 2% (OR = 1.02, 95% CI: 1.01–1.63). Furthermore, we transformed the METS-IR from a continuous variable into a categorical variable (quartiles) for sensitivity analysis (**Table 3**). In Model 1 (not adjusted), the risk of HF in the highest quartile increased by 1.79 compared with the lowest quartile (OR: 2.79, 95% CI 2.12, 3.65, p < 0.001). The OR (95% CI) in Model 2 was 2.93 (2.21, 3.88) after adjusting for age, gender, and race. Model 3 was adjusted for covariates in Model 2 plus education level, marital status, smoking status, alcohol drinking status, hypertension, high cholesterol, CHD, angina, heart attack, stroke, and diabetes status, and the OR for HF increased with a higher METS-IR, showing a significant association (OR: 1.73, 95% CI 1.25, 2.39, p < 0.001). There was statistical significance in the test for trend in Model 1, 2, and 3. After adjusting for all covariates in Model 4, the fully adjusted ORs and 95% CIs of Q2, Q3 and Q4 compared with Q1 were 0.73 (0.51, 1.06), 0.80 (0.54, 1.19) and 1.11 (0.69, 1.78) (p for trend = 0.475), respectively.

**Table 3:** Association of METS-IR with heart failure in different models among all participants.

METS-IR	Model 1	P value	Model 2	P value	Model 3	P value	Model 4	P value
	OR (95%CI)		OR (95%CI)		OR (95%CI)		OR (95%CI)	
Per 1 increment	1.03 (1.02, 1.04)	<0.001	1.04 (1.03, 1.04)	<0.001	1.03 (1.02, 1.03)	<0.001	1.02 (1.01, 1.03)	0.002
Quartile								
Q1	1		1		1		1	
Q2	1.21 (0.88, 1.65)	0.235	1.02 (0.74, 1.40)	0.903	0.84 (0.59, 1.18)	0.313	0.73 (0.51, 1.06)	0.102
Q3	1.68 (1.26, 2.26)	0.001	1.40 (1.03, 1.88)	0.029	1.04 (0.74, 1.45)	0.839	0.80 (0.54, 1.19)	0.271
Q4	2.79 (2.12, 3.65)	<0.001	2.93 (2.21, 3.88)	<0.001	1.73 (1.25, 2.39)	0.001	1.11 (0.69, 1.78)	0.679
P for trend		<0.001		<0.001		<0.001		0.475

Model 1: no covariates were adjusted;

Model 2: adjusted for age, gender, race;

Model 3: adjusted for covariates in Model 2 plus education level, marital status, smoking status, alcohol drinking status, hypertension, high cholesterol, CHD, angina, heart attack, stroke, and diabetes status;

Model 4: adjusted for covariates in Model 3 plus PIR, PA, SBP, DBP, BUN, serum creatinine, eGFR, Serum uric acid, HbA1c, Hb, TC, LDL, waist circumference.

CHD, coronary heart disease; PIR, family poverty income ratio; PA, physical activity; SBP, systolic blood pressure; DBP, diastolic blood pressure; BUN, blood urea nitrogen; eGFR, estimated glomerular filtration rate; HbA1c, hemoglobin A1c; Hb, hemoglobin; TC, total cholesterol; LDL-C, low-density lipoprotein cholesterol; OR, odds ratio CI, conﬁdence interval.

### 3.3 Non-linearity and threshold effect analysis between METS-IR and HF

**Figure 2:**
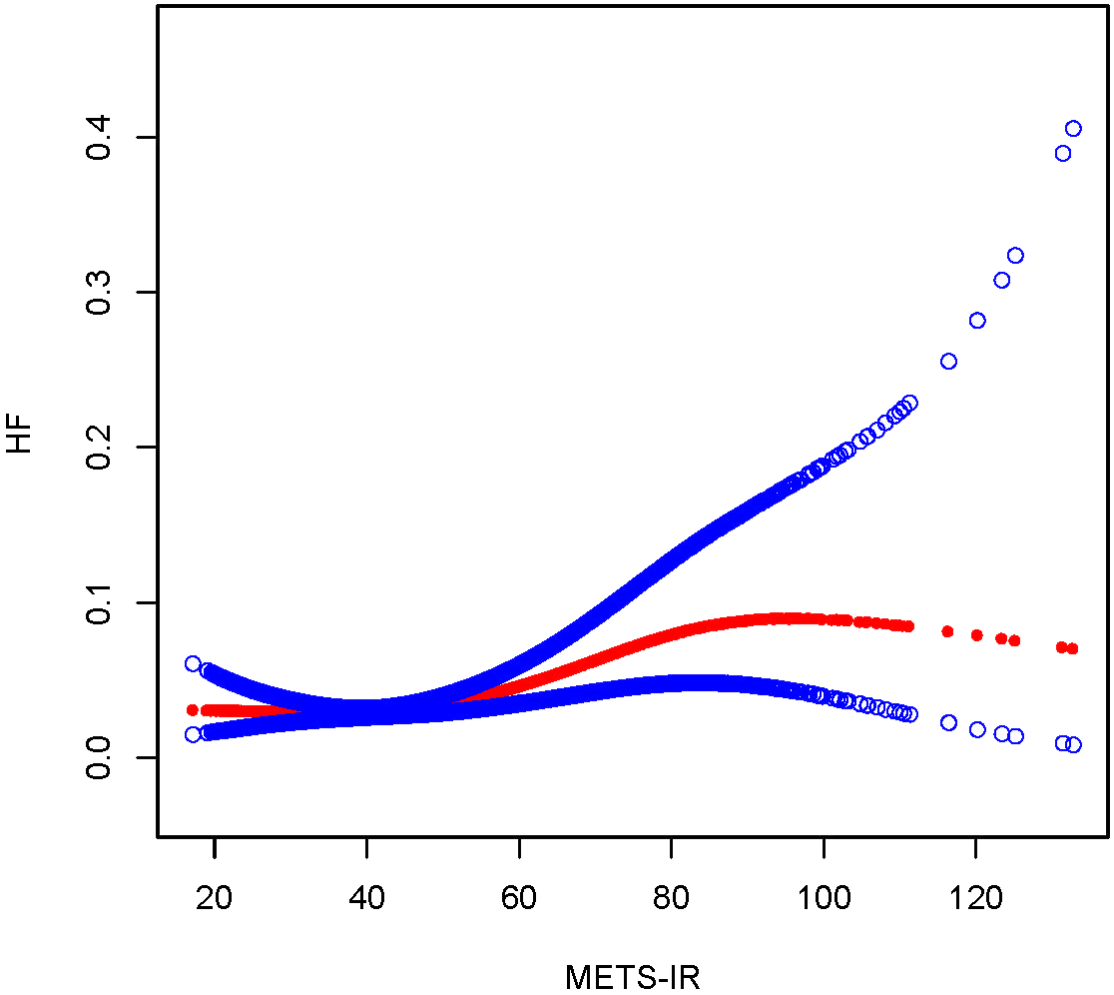
The smooth curve fit for the association between METS-IR and the prevalence of heart failure.

**Figure 3:**
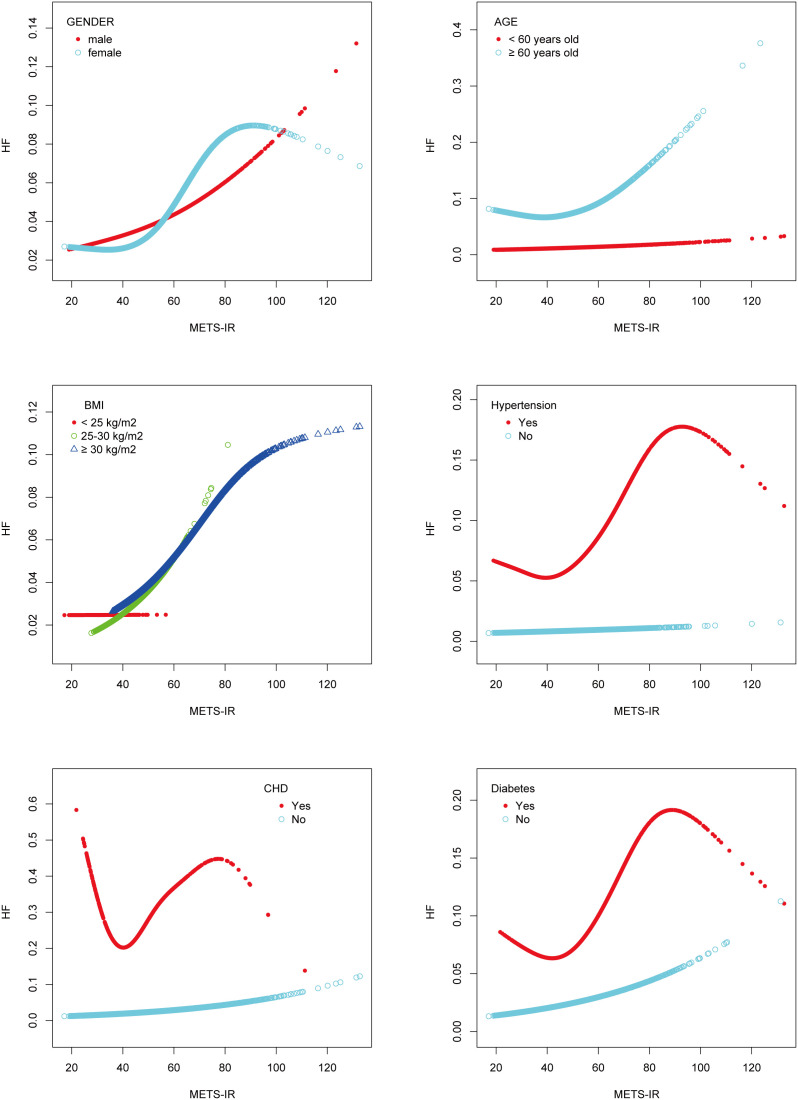
Subgroups analysis for the association between METS-IR and the prevalence of heart failure by gender, age, BMI, hypertension, CHD, and diabetes.

To illustrate the nonlinear relationship and saturation effect between METS-IR and HF, a smooth curve fitting technique was employed, as shown in **Figures 2** and **3**. Among all participants, the correlation between METS-IR and HF exhibited a J-shaped curve after adjustments, with inflection points observed at 40.966 (**Table 4**). When the METS-IR measurements were below 40.966, the effect values were not statistically significant. However, when the METS-IR exceeded 40.966, there was a significant effect value of 1.025.

**Table 4:** Threshold effect analysis of METS-IR index and HF.

METS-IR	Adjusted OR (95% CI), *P*-value
Fitting by the standard linear model	1.020 (1.007, 1.034) 0.002
Fitting by the two-piecewise linear model	
Inflection point	40.966
METS-IR < 40.966	0.982 (0.951, 1.014) 0.274
METS-IR ≥ 40.966	1.025 (1.012, 1.039) <0.001
P for Log-likelihood ratio	0.014

Adjusted for age, gender, race, education level, marital status, smoking status, alcohol drinking status, hypertension, high cholesterol, CHD, angina, heart attack, stroke, and diabetes status, PIR, PA, SBP, DBP, BUN, serum creatinine, eGFR, Serum uric acid, HbA1c, Hb, TC, LDL, waist circumference.

Additionally, the study was carried out using smoothing curve fitting stratified by gender, age, body mass index, hypertension, CHD, and diabetes in order to confirm whether the positive correlation between METS-IR and HF was non-linear across various cohort characteristics (**Figure 3**). The final results indicated that the METS-IR association with HF was curvilinear in female, aged ≥ 60 years, BMI <25 kg/m^2^, hypertension, CHD, and diabetes.

### 3.4 Subgroup analysis

**Figure 4:**
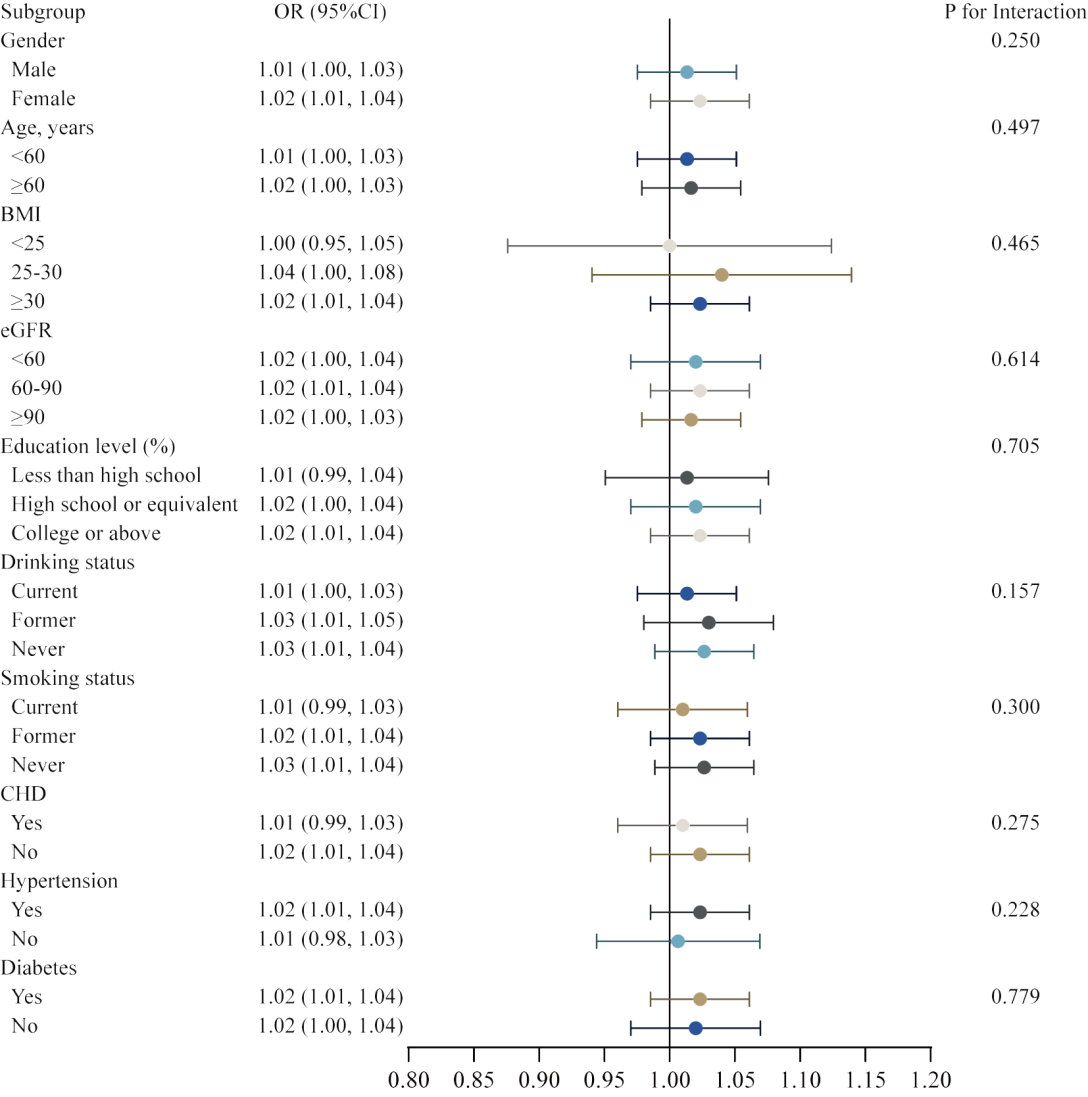
Subgroup analysis for the association between METS-IR and heart failure.

Subsequently, we conducted a subgroup analysis with interaction test to investigate whether the association between METS-IR and the risk of HF was robust in various demographic settings. In Model 4, all covariates were taken into account, with the exception of the covariates that were utilized to establish subgroups. The robustness of our main finding between the METS-IR and gender, age, BMI, eGFR, education level, drinking status, smoking status, CHD, hypertension, and diabetes was illustrated in the figure, with no significant interactions and all P for interaction were > 0.05 (**Figure 4**).

### 3.5 ROC analysis

**Figure 5:**
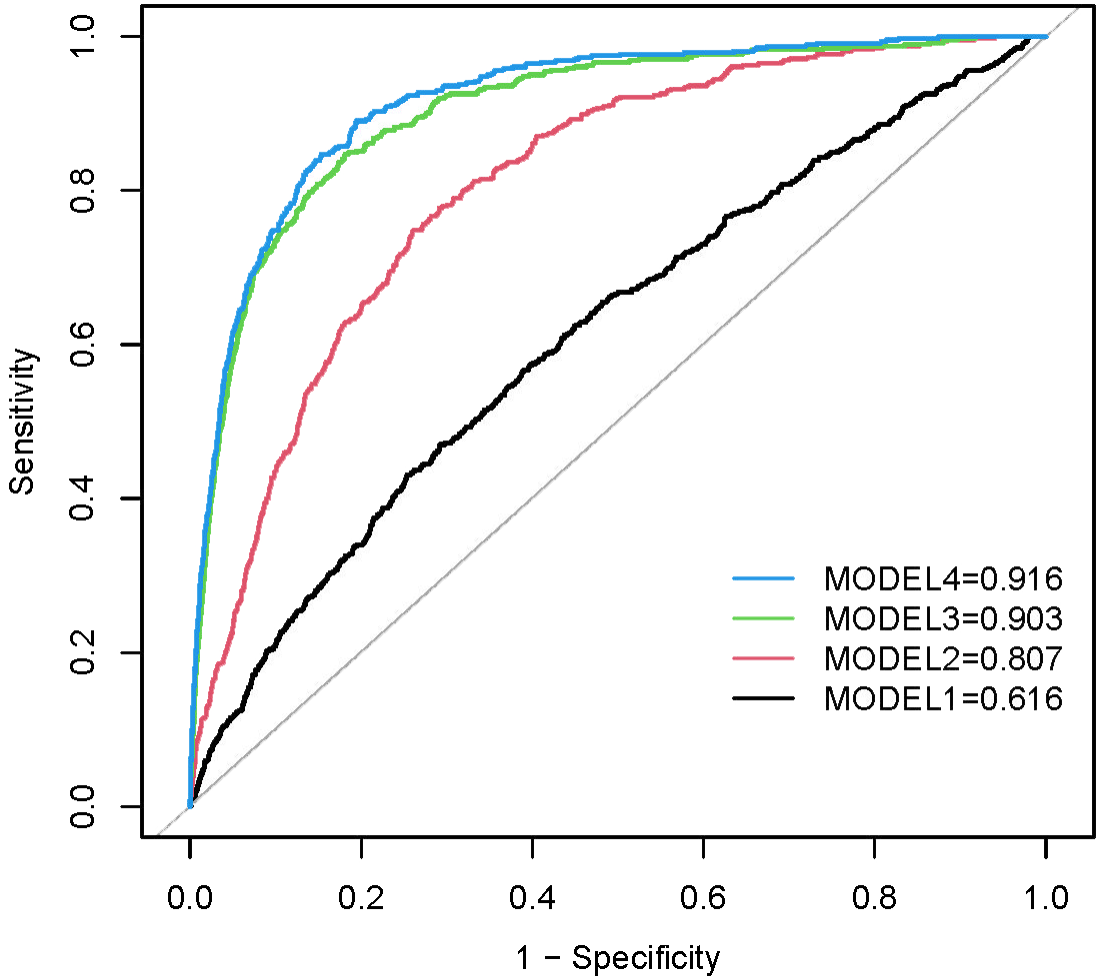
ROC curves between METS-IR and HF in Model 1, Model 2, Model 3, and Model 4. Model 1 was unadjusted. Model 2 was adjusted for age, gender, and race. Model 3 was adjusted for covariates in Model 2 plus education level, marital status, smoking status, alcohol drinking status, hypertension, high cholesterol, CHD, angina, heart attack, stroke, and diabetes status. Model 4 was adjusted for covariates in Model 3 plus PIR, PA, SBP, DBP, BUN, serum creatinine, eGFR, Serum uric acid, HbA1c, Hb, TC, LDL, waist circumference.

The receiver operating characteristic curves (ROC) for METS-IR to detect heart failure in four models are presented in **Figure 5**. METS-IR (continuous) predicted the area under the ROC curve of 0.616 (95% CI: 0.589–0.642, p < 0.001) in the unadjusted model. The optimal cutoff value was 48.11, with a sensitivity of 47.0% and a specificity of 70.8%. Statistical significances were found in predicting the area of the ROC curves in Model 2 (AUC: 0.807, 95% CI: 0.789–0.825, p < 0.001), Model 3 (AUC: 0.903, 95% CI: 0.889–0.917, p < 0.001), and Model 4 (AUC: 0.916, 95% CI: 0.904–0.928, p < 0.001).

## 4 Discussion

The results of this large cross-sectional study indicate that METS-IR and the risk of heart failure were positively correlated after adjusting for potential confounders. These connections were similar across populations, according to the findings of subgroup analyses and interaction testing. Notably, a non-linear relationship was observed between METS-IR and HF risk, with a distinct inflection point occurring at a METS-IR measurement of 40.966, characterized by a J-shaped curve. METS-IR may be a significant predictor of heart failure and a potential therapeutic target.

In comparison to other non-insulin-based indices, recent research has demonstrated that METS-IR is a viable and trustworthy alternative biomarker for IR. As a valid surrogate for insulin sensitivity that includes both laboratory and anthropometric measurements, METS-IR has been proposed as a tool to identify individuals at an elevated risk of developing T2DM, metabolic syndrome, and other cardiovascular diseases (CVDs) at an early age (16, 18). To the best of our knowledge, our study is the first to examine the link between METS-IR and HF. Previous studies have investigated the correlation between METS-IR and cardiovascular risk factors and CVD in participants with or without diabetes. Han et al. conducted an analysis on a cohort of 15,453 normoglycemic participants and found that METS-IR was a significant risk indicator for pre-hypertension (pre-HTN) and hypertension (HTN). Their findings indicated that for each unit increase in METS-IR, there was a 7% rise in the prevalence of pre-HTN (adjusted odds ratio [OR] = 1.07, 95% confidence interval [CI]: 1.06–1.08) and a 13% increase in the prevalence of HTN (adjusted OR = 1.13, 95% CI: 1.10–1.16) (19). A stronger positive correlation between METS-IR and pulse wave velocity (PWV) was observed in individuals with high cardiovascular risk conditions after adjusting for sex, age, treatment for hypertension, and smoking status (β=0.350, 95% CI: 0.204–0.418), compared to other non-insulin-based IR indices such as the TG/HDL-C index and the TyG index (20). Another long-term prospective cohort involving 14220 individuals diagnosed with H-Type Hypertension in China, with a median follow-up of 3.94 years, found that METS-IR was not only significantly positively associated with the development of CVD (HR=1.66, 95% CI: 1.30–2.12), but also with an increased risk of cardiovascular mortality (HR=1.57; 95% CI, 1.22–2.02) and all-cause mortality (HR=1.33; 95% CI, 1.11–1.60) (21). Furthermore, another longitudinal cohort study involving 17,943 Korean participants without diabetes found that elevated METS-IR was positively and independently associated with the incidence of ischemic heart disease (IHD). During a 50-month follow-up period, HRs of IHD for METS-IR quartiles 1–4 were 1.00, 1.62 (95% CI 1.04–2.53), 1.87 (95% CI 1.20–2.91), and 2.11 (95% CI 1.35–3.30) after adjusting for potential confounding variables (15). However, the evidence for the association between METS-IR and heart failure among the general adult population in the United States is limited. Based on a well-designed national survey, our study first demonstrated that METS-IR was significantly higher in the heart failure group than in the non-heart failure group. Furthermore, higher METS-IR predicted a higher risk of heart failure, which was consistent with the adverse cardiovascular health effects of METS-IR described in previous research (15, 31). Analysis of the smoothed curve fit revealed a J-shaped relationship between METS-IR and heart failure, with an inflection point at 40.966. By being able to identify the threshold value at which the risk of heart failure begins to increase, physicians are able to target specific patient subpopulations for closer monitoring and intervention. This personalized approach to heart failure management can provide a new foundation for the prevention and control of heart failure, leading to improved patient outcomes and reduced healthcare costs. Furthermore, subgroup analysis indicates that the relationship between METS-IR and HF is not potentially modified by variables such as gender, age, BMI, hypertension, coronary heart disease, or diabetes.

Several potential mechanisms may explain this positive correlation between METS-IR and HF. Accumulating evidence confirms that IR plays a crucial role as a risk factor for HF. First, IR can cause energy metabolism in the myocardium, resulting in a shift towards using fatty acid metabolism rather than glucose for myocardial energy acquisition. One way that the degree of IR affected the heart was by reducing its capacity to utilise fatty acids, which can result in the accumulation of lipids and an elevation in their concentration. Ultimately, this will result in the occurrence of HF due to mitochondrial dysfunction and apoptosis (22). Conversely, in diabetes, elevated reactive oxygen species (ROS) are produced as a result of progressive mitochondrial impairment. This exacerbates diabetic cardiomyopathy (DCM) and worsens oxidative stress, further impairing cardiac function (23). Furthermore, the presence of excess circulating free fatty acids can lead to the development of lipotoxicity, which in turn can result in the impairment of pancreatic and myocardial β-cell function, as well as glucose intolerance and heart failure (24). Second, the nucleotide-binding oligomerization domain-like receptor family, pyrin domain-containing 3 (NLRP3), can be activated by IR, which results in the production of interleukin-18 (IL-18) and interleukin-1 beta (IL-1β), as well as local tissue inflammation. Studies have shown that therapeutic interventions involving silencing of the NLRP3 gene have been effective in alleviating cardiac inflammation, pyroptosis, and fibrosis, as well as improving cardiac function (25, 26). Third, calcium plays a crucial role in regulation of mitochondrial function. However, cardiac insulin resistance impairs calcium homeostasis via the PKB–SPEG–SERCA2a pathway, which can diminish the cardiac capacity to process calcium (27) and impair cardiac diastolic function (28), and contributes to the development of DCM (29). Fourth, IR and the resulting hyperinsulinemia could lead to an increase in blood pressure by activating the sympathetic nervous system and the renin-angiotensin-aldosterone system, ultimately resulting in myocardial fibrosis and cardiac dysfunction (30).

Conversely, there is evidence that extremely low levels of TG, FPG, or BMI have a negative impact on health and may even accelerate the onset of certain diseases. Notably, lower IR levels were associated with lower fasting glucose levels. It has been demonstrated that hypoglycemia and rapid fluctuations in blood glucose levels increase levels of counter-regulatory hormones such as epinephrine and norepinephrine. These hormones have the potential to cause platelet aggregation and vasoconstriction, which would accelerate the development of ischaemia in the cardiovascular system (31). A J-shaped relationship between blood glucose levels and cardiovascular events or all-cause mortality was confirmed by a study, with lower fasting blood glucose levels being associated with an increase in adverse events (32). Similarly, low TG levels were identified as an elevated risk of hemorrhagic stroke (33), cardiovascular outcomes and all-cause mortality in patients with type 2 diabetes (34). Previous studies have shown that low BMI is inversely related to several common risk factors for atherosclerosis. However, some large Western population studies have shown a U-shaped association between BMI and CVD mortality (35). According to studies, BNP and NT-proBNP concentrations are inversely correlated with BMI, and particularly high levels have been reported in patients with cardiac cachexia. Low BMI has been linked to a worse prognosis in patients with chronic heart failure, and survival is further impaired if CHF progresses to cardiac cachexia (36). The causality of low body weight and poor prognosis is debated, with some arguing that it is a consequence and others suggesting that it is a cause, but there’s no denying that the obesity paradox exists.

The main advantage of this study is that it was conducted using data from the NHANES, which were collected using a stratified multistage probability sampling strategy. Our study has a large sample size with 14,772 enrolled participants, which increases the representativeness and reliability of the study. Furthermore, we adjusted for potential confounding variables, including age, gender, race, education level, marital status, smoking status, drinking status, hypertension, high cholesterol, CHD, angina, heart attack, stroke, and diabetes status, PIR, PA, SBP, DBP, BUN, serum creatinine, eGFR, serum uric acid, HbA1c, Hb, TC, LDL-C, and waist circumference to reduce the impact of confounding factors and obtain more reliable results. The relationship between METS-IR and heart failure among US adults was initially investigated, revealing a J-shaped association with an inflection point at 40.966. The inflection point identified in our study serves to further enhance the clinical utility of METS-IR measurements.

It should be noted that there are some limitations to our study. First, due to the cross-sectional design of NHANES, we were unable to determine a causal relationship between METS-IR and heart failure. Second, despite our efforts to account for potential confounding variables, the influence of unidentified or unmeasured confounders could not be completely ruled out. However, the existing association was robust enough that it was unlikely to be significantly altered by unaccounted for confounding variables. Third, the NHANES database does not provide relevant questionnaire in this database that mentions the classification of heart failure, as well as brain natriuretic peptide, ultrasound and imaging studies such as cardiac ultrasound and cardiac magnetic resonance that reflect the details information of heart failure. Therefore, this study cannot further evaluate the relationship between METS-IR and the severity and classification of heart failure. Fourth, it is important to note that the study population is limited to the United States. Therefore, further prospective cohort studies are necessary to confirm and promote the current findings in a larger population.

## 5 Conclusions

In summary, METS-IR was significantly associated with the risk of HF positively. This cross-sectional study revealed that METS-IR had a J-shaped relationship with the risk of HF among a nationally representative sample of adults in the United States. The METS-IR cut-off (40.966) has a certain clinical application value. However, more longitudinal studies are still needed to further verify causal relationships and validate the results in different classifications of heart failure populations.

## Data availability statement

The datasets presented in this study can be found in online repositories. The names of the repository/repositories and accession number(s) can be found below: https://www.cdc.gov/nches/nhanes.

## Author contributions

XS: Writing – review & editing, Writing – original draft, Visualization, Validation, Software, Methodology, Formal analysis, Data curation, Conceptualization. CZ: Writing – review & editing, Supervision. XZ: Writing – review & editing, Supervision, Methodology.
